# Long-Range Correlations of Global Sea Surface Temperature

**DOI:** 10.1371/journal.pone.0153774

**Published:** 2016-04-21

**Authors:** Lei Jiang, Xia Zhao, Lu Wang

**Affiliations:** 1 School of Marine Sciences, Nanjing University of Information Science and Technology, Nanjing, China; 2 Jiangsu Research Center for Ocean Survey Technology, Nanjing, China; 3 Laboratory of Ocean Circulation and Waves, Institute of Oceanology, Chinese Academy of Sciences, Qingdao, China; 4 Nuclear and Radiation Safety Center, Ministry of Environmental Protection, Beijing, China; CNRS, FRANCE

## Abstract

Scaling behaviors of the global monthly sea surface temperature (SST) derived from 1870–2009 average monthly data sets of Hadley Centre Sea Ice and SST (HadISST) are investigated employing detrended fluctuation analysis (DFA). The global SST fluctuations are found to be strong positively long-range correlated at all pertinent time-intervals. The value of scaling exponent is larger in the tropics than those in the intermediate latitudes of the northern and southern hemispheres. DFA leads to the scaling exponent **α** = 0.87 over the globe (60°S~60°N), northern hemisphere (0°N~60°N), and southern hemisphere (0°S~60°S), **α** = 0.84 over the intermediate latitude of southern hemisphere (30°S~60°S), **α** = 0.81 over the intermediate latitude of northern hemisphere (30°N~60°N) and **α** = 0.90 over the tropics 30°S~30°N [fluctuation *F*(*s*) ~ *s*^**α**^], which the fluctuations of monthly SST anomaly display long-term correlated behaviors. Furthermore, the larger the standard deviation is, the smaller long-range correlations (LRCs) of SST in the corresponding regions, especially in three distinct upwelling areas. After the standard deviation is taken into account, an index *χ* = *α* * *σ* is introduced to obtain the spatial distributions of *χ*. There exists an obvious change of global SST in central east and northern Pacific and the northwest Atlantic. This may be as a clue on predictability of climate and ocean variabilities.

## Introduction

The ocean plays an important role in the complicated climate system. The huge thermal capacity in the ocean enables SST variability to exhibit strong persistence characteristics. Sea Surface Temperature (SST) as a key factor that the ocean is connected with climate on a global scale is not always easy to analyze due to the nonlinear and irregular evolutions with different spatio-temporal scales. To characterize Long Range Correlations (LRCs) of the SST fluctuation on all pertinent temporal scales still poses a challenge. Therefore, it is necessary to detect spatio-temporal evolutions of LRCs in the global SST fluctuation.

In recent years, long-range persistence or dependence has been investigated in many fields. DFA developed by Peng et al. [1] has been established as an important tool to detect LRCs in time series with non-stationarity. Compared to the traditional approaches, such as the power-spectrum and correlation analysis, DFA can systematically eliminate trends in the data and thus reveal intrinsic dynamic properties such as scaling behaviors often masked by non-stationarities [1–7]. DFA has been successfully applied to evaluate characteristics of data sets such as long-time temperature records [8–18], financial time-series [19], heart rate dynamics [20], air pollution [21], ozone variations [22], regional temperature records [23–24], global surface air temperature [25], among others. Previously, it was observed that the exponent in temperature records had roughly the same value of 0.65 for the continental [15].

LRCs consist in the temporal evolution of different climatic sub-systems generated by natural and anthropogenic causes and keep significant power-law correlation behaviors over a wide time scales [26]. In other words, the interactions of different climate sub-systems are non-stationary and even non-linear processes. LRCs characterize the scaling behaviors of various parameters with all pertinent spatial-temporal scales. The DFA method can handle the nonstationary of the process with trend. The diagnosis and prediction of the mechanisms are of great importance to descript the temporal evolution of different variables. Therefore, LRCs and geographical distributions of SST time series are fundamental to further understand climate change and air sea interaction under different backgrounds. Moreover, it may provide a valid basis to test existing and future climate and ocean models, especially for different regions in the world.

In the study of SST fluctuations, Monettia et al. [14] noticed that the fluctuations of SST in the Atlantic and Pacific oceans display a non-stationary behavior at short-time scales that seems to end at 10 months, while a stationary behavior above time scales of 10 months. This reveals the LRCs of SST. Zhu et al. [27] analyzed spectrum and scaling of meridional overturning circulation (MOC) in the Atlantic ocean. The power-law scaling in the spectra is *S* (ƒ)~ƒ^-β^ for lowest frequencies. LRCs are found in these spectra when the exponent β is larger than 0. Gan et al. [28] used the optimum interpolation sea surface temperature data to analyze scaling behaviors of SST in the South China Sea. They think the time interval of LRCs spreads from about 1 month to 4.5 yr over a wide period and LRCs depend on different geographic locations. Alvarez-Ramirez et al. [29] found that there exist LRCs and multi-fractal characteristics in continental and oceanic monthly temperatures for both Northern and Southern hemispheres. Moreover, the persistence of ocean temperatures exhibits a cyclic behavior around an average value of 22 years. Luo et al. [30] studied scaling behaviors of SST in globe divided into two pronounced regimes by taking into the ENSO consideration as a general crossover. There exist non-stationary and anti-persistent behaviors for SST at the small-scale, while stationary and LRCs at the large-scale. Zhang and Zhao [31] revealed asymmetric LRCs of SST in globe with upward and downward analysis using asymmetric detrended fluctuation analysis (A-DFA) method. The LRCs of SST takes on a letter ‘‘V” in the tropical Pacific ocean, where there exist the larger scaling exponents at two sides of the eastern tropical Pacific. Such pattern may be affected by ENSO which the period is 2~7 years in middle and east tropical Pacific.

The main aim of this paper is to detect LRCs and the geographical distribution of the scaling law of global SST fluctuation and to discuss if it exhibits positive LRCs at different time scales using DFA. The estimation of the power-law exponent **α** in the global SST data sets is outlined.

## Method and Data

### Data Records

The Met Office Hadley Centre's monthly sea ice and sea surface temperature (HadISST) data set is a combination of globally SST and sea ice fields focused on a 1 degree latitude-longitude grid from January 1870 to December 2009. The HadISST data set replaces the Global sea Ice and Sea Surface Temperature (GISST) data sets and merges monthly SST from the Comprehensive Ocean-Atmosphere Data Set (COADS) to enhance the data coverage [32]. We accessed the data from http://www.metoffice.gov.uk/hadobs/hadisst/data/download.html. The annual cycles from the raw data *T*_*i*_ are removed by computing the SST anomaly Δ*T*_*i*_ = *T*_*i*_ − 〈*T*_*i*_〉_*m*_, where 〈*T*_*i*_〉_*m*_ denotes the average value for a given month.

The monthly SST anomaly during 1870–2009 is used to explore the temporal scaling behavior over the globe. Varotsos et al. [25] separated global surface air temperature anomalies into three regions the Northern Hemisphere (NH), Southern Hemisphere (SH) and globe to investigate the existence of LRCs in their temporal evolution. For that reason, six areas are divided into the tropics (30°S -30°N), the intermediate latitude of NH (30°N-60°N), the intermediate latitude of SH (30°S-60°S), NH (0°N-60°N), SH (0°S-60°S), and globally area. In the present study, the results are calculated for all the grids in all time-intervals.

### The DFA Method

First, let us briefly describe some important steps of the DFA method. (1) The anomaly time series (with *N* samples) are integrated to obtain the so-called profile $y(i)=\sum_{k=1}^{i}\Delta T_{k}$. (2) The profile is divided into non-overlapping segments of equal length *s*, indexed by k = 1,…, *N*_*s*_ with *N*_*s*_ = [*N* / *s*]. Since the record length *N* is not always a multiple of the segment length *s*, a remainder often exists at the end of the profile. In order to keep this part of the record, the same procedure is repeated as a beginning from the other end of the record. Therefore, 2*N*_*s*_ segments are obtained altogether. (3)The local trend for each segment is calculated by a least-square fit *y*_*s*_(*k*). (4) The profile is detrended by subtracting the local fit and the fluctuation function for each segment length *s* is calculated by $F(s)=\sqrt{\frac{1}{2N_{s}}\sum_{k=1}^{2N_{s}}{\left[y(k)-y_{s}(k)\right]}^{2}}$, which is the mean of the variances of the profile with respect to the fits over all segments. Typically, *F*(*s*) will increases with *s*. A linear relationship on a log-log plot indicates the presence of power-law scaling *F*(*s*) ~ *s*^*α*^. The value of exponent *α* represents the degree of the correlation in the signal: if *α* = 0.5, the signal is uncorrelated (white noise); if *α* > 0.5, the signal is correlated; if *α* < 0.5, the signal is anti-correlated; for *α* = 1, the signal is 1/f noise. Different orders *m* of DFA (DFA1, DFA2, etc.) differ in the order of the polynomials used in the fitting procedure [for more details, see Kantelhardt et al. [3].

## Results and Discussion

**Fig 1:**
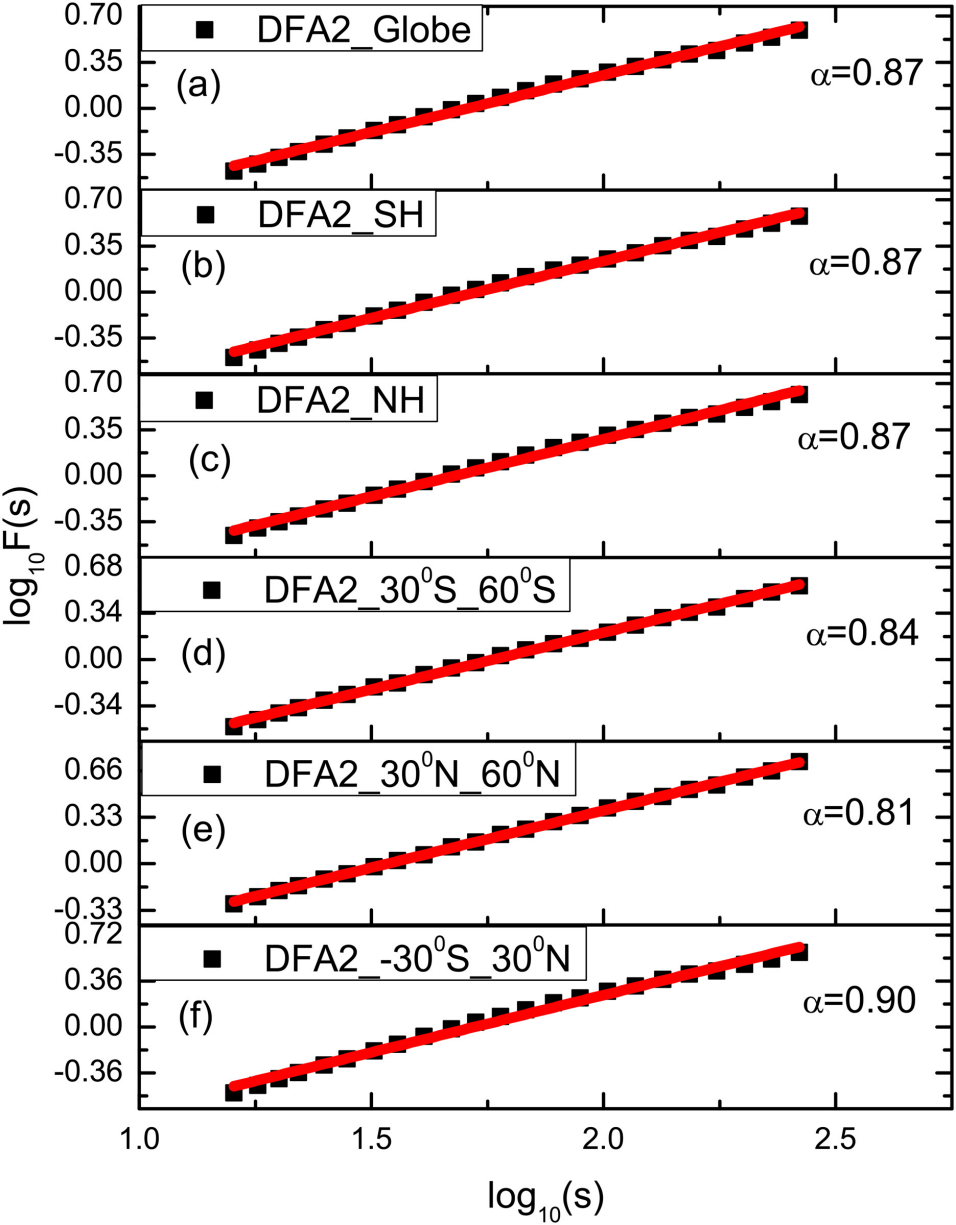
Log-log plots of power-law relationship between the detrended variability *F*(*s*) and the time scale *s* in the global zones, southern hemisphere, north hemisphere, the middle latitude zones, and the tropics (solid squares for the SST series (annual cycles are removed) using DFA2 and red solid lines for the records represent linear fit of SST fluctuations).

The results obtained from the application of the DFA2 method to the global SST time series in the different latitude belts are depicted in Fig 1a, 1b, 1c, 1d, 1e and 1f. The values of scaling exponent are **α** = 0.87 over the globe, NH, and SH, **α** = 0.84 over the intermediate latitude of SH, **α** = 0.81 over the intermediate latitude of NH and **α** = 0.90 over the tropics, respectively. It is found that the value of scaling exponent in the tropics is higher than those in other regions. The values of scaling exponent in globally, NH and SH are higher than those in intermediate latitudes. There exists dynamical memory for global SST fluctuations in the globe for all time scales. The curves are approximately straight lines and their slopes are used as better representations of LRCs. Actually, all six zones have similar characteristics. The strong persistence characteristics means that the global SST fluctuations are positively long-range power-law correlated from small time intervals up to 139 years. Fraedrich and Blender [33] analyzed in observations and simulation and found the value of scaling exponent α = 1 over the oceans, while our results show that the power-law scaling in the fluctuations of monthly SST anomalies varies from different geographical distributions. The value α = 0.90 in the tropics is highest than those at other regions, even around 1 which agrees with the study by Blender and Fraedrich [16]. However, the difference of LRCs is obvious in the region of intermediate latitude. Luo et al. [30] studied the scaling behaviors of SSTA in different regions, where the value of scaling exponents as a whole are high in the extra-tropical regions, but low in the tropical regions. By comparison, the value α = 0.87 globally in this paper is higher than that α = 0.78 obtained by Luo et al. [30]. This implies that there exists stronger long-term memory for monthly SST anomalies. Moreover, the fluctuations of SST in the intermediate latitudes of the northern and southern hemispheres display persistence behaviors and LRCs at all pertinent scales that seem to end at 300 months. Moreover, this result suggests that the classical Markov-type stochastic theory does not apply to the long-term correlations between the fluctuations in the global SST variability. In fact, the global SST fluctuations exhibit more slowly decaying correlations.

**Fig 2:**
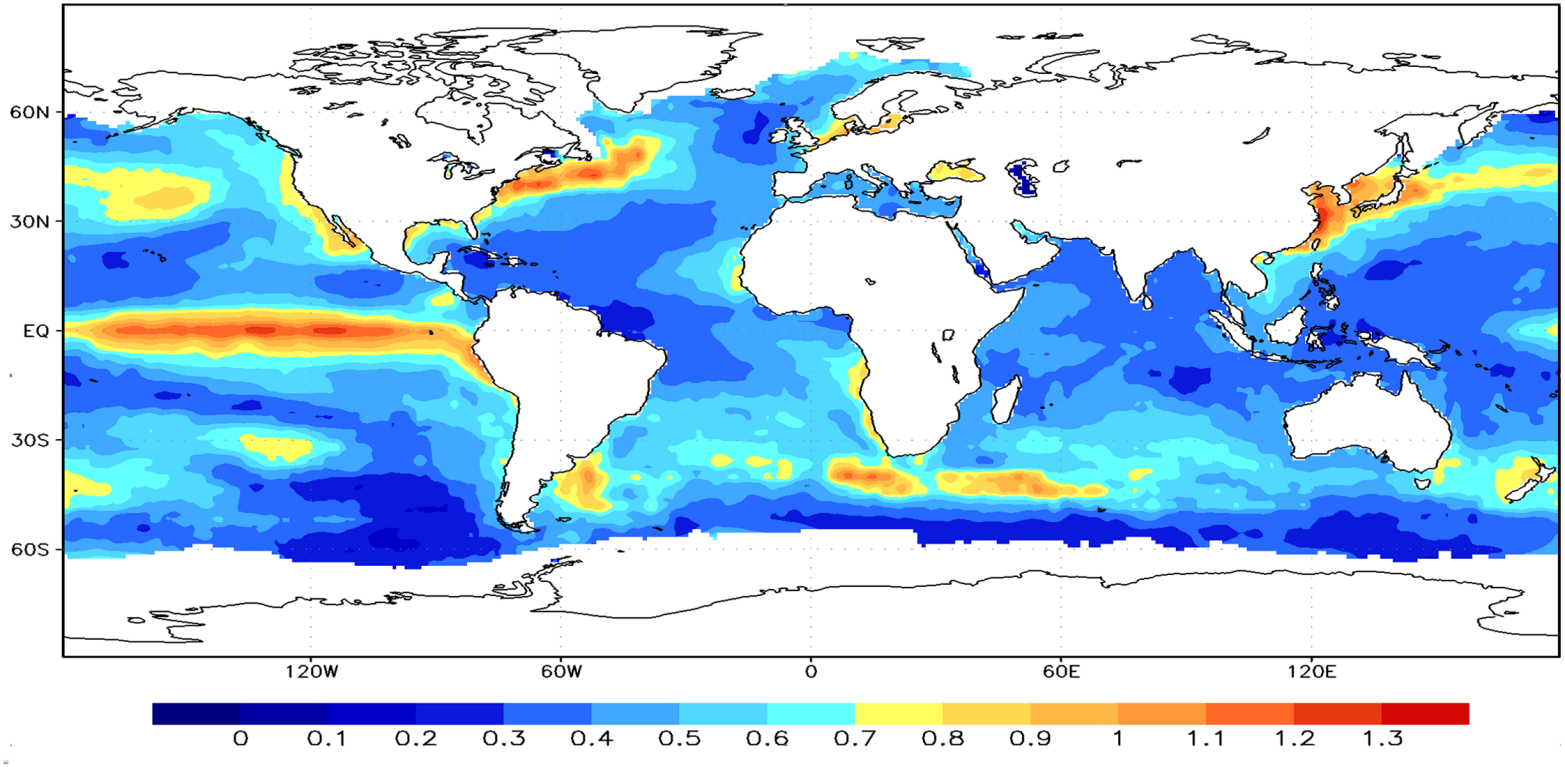
Spatial distributions of the standard deviation in the global SST anomaly time series during the time 1870–2009.

In order to characterize the magnitude of SST variability, it is necessary to analyze the departure of the SST from the monthly average value, which is defined as standard deviation. The values of standard deviation reflect variation extents of the deviating mean value. Standard deviation of variables as an important consulting indicator determines the environment conditions in different zones. The spatial distribution of standard deviation in the global monthly SST anomalies records are exhibited in Fig 2 during 1870–2009.

The maximum standard deviation of monthly SST anomaly in the tropics was found in the equatorial central east Pacific, while standard deviations are about 0.7°C–1°C in other three notable regions of the extra-tropics. The trade winds are one of important factors to affect the variability of SST in the central Pacific. The easterly weak Pacific trade winds in tropical regions enhance the warming of the surface ocean and an intensification of the subsurface thermocline under transient global warming. However, the anomalous strong trade winds in tropical Pacific areas accelerate the equatorial current and countercurrent [34]. Furthermore, upwelling governed by the interaction between atmospheric and oceanic processes is also an important mechanism to affect the standard deviation of SST [35]. There exists a distinct upwelling area in the equatorial central east Pacific zone [36–37]. Moreover, the wind stress at the ocean surface brings geostrophic currents, where colder water of upwelling from land sinks under lighter water [38]. Meantime, the coastal Benguela eastward current, the Labrador current and the Kuroshio current play important roles on the impact of climate change considering the global meridional overturning circulation. Sea waters in these areas are modified by local mixing and air–sea interaction and then affect SST. SST anomalies provides the long-term memory in the climate system. The values of standard deviation are around 0.2°C–0.4°C and relatively low in the western Pacific and Atlantic and the Southern ocean [39–40]. Large standard deviation reflects the complicated conditions of the SST fluctuations and variations in certain extents. In fact, the large value of standard deviation increases the probability of extreme events for the monthly SST anomaly records.

**Fig 3:**
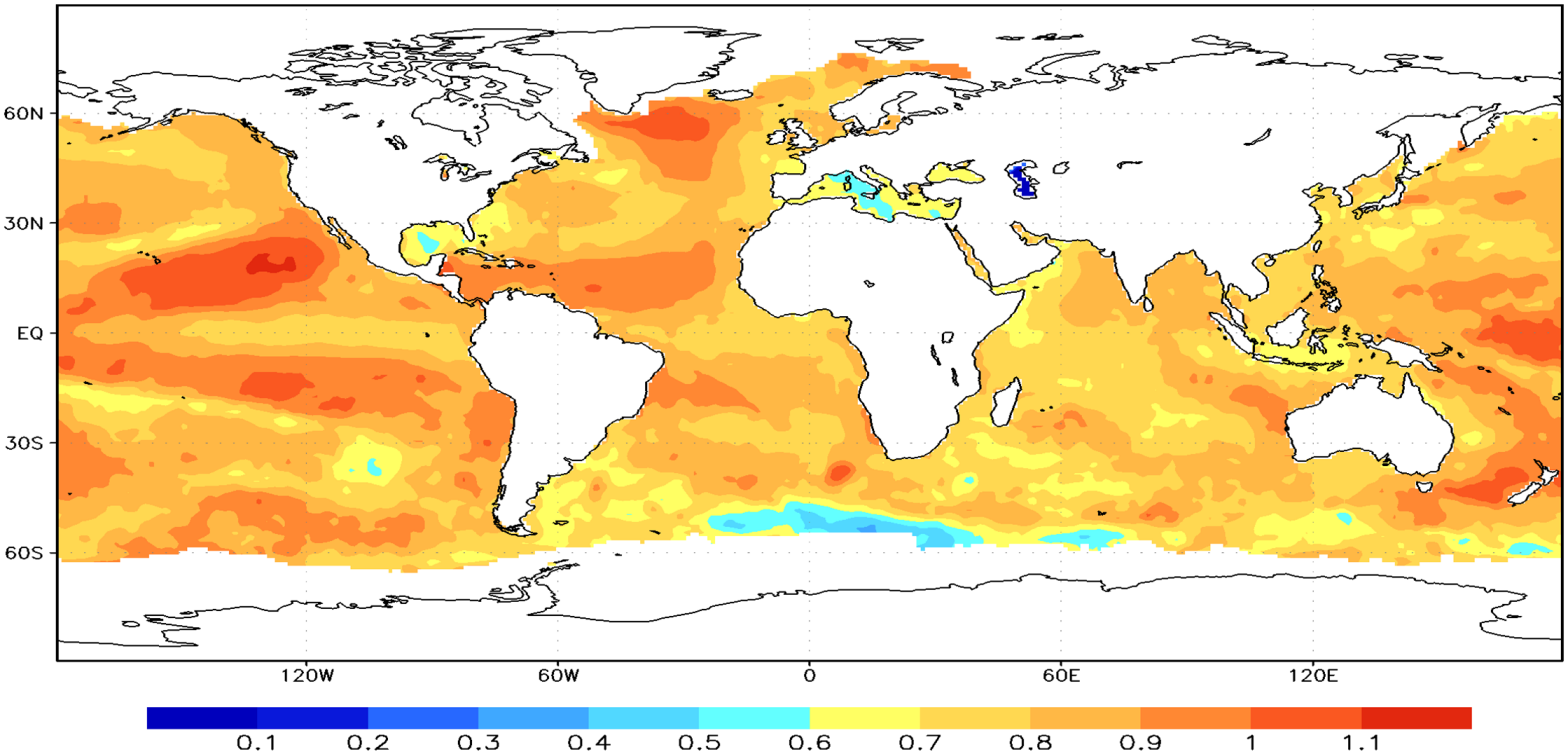
Spatial distributions of the scaling exponents in the global SST anomaly time series during the time 1870–2009 employing DFA2.

Next, the geographical distributions of the scaling exponents of the global SST fluctuation are analyzed by employing the DFA2 method, as shown in Fig 3. It is found the fluctuation exponents are different in different regions. The values of scaling exponent in the eastern tropical Pacific are low indicating that scaling behaviors may be affected by the ENSO phenomenon [31, 41]. The value of scaling exponent is close to 1 for North and South Pacific. This indicates that SST anomaly exhibits a strong long-term memory in both sides of the tropical Pacific near the equator, which is consistent with the results [36]. Comparing with the spatial distribution of standard deviation, it is found that LRCs are weak when standard deviation is large in those regions, especially in three distinct upwelling areas mentioned above. Large fluctuations of SST increase the probability of occurrence of extreme value, but reduce its long-term memory and predictability [42]. However, the value of scaling exponent is larger than 0.6 over the global oceans implying positive LRCs as a whole. Different spatial distributions in exponents are possibly modulated by ENSO, Pacific Decadal Oscillation (PDO) and Atlantic Multidecadal Oscillation (AMO) [43–45].

**Fig 4:**
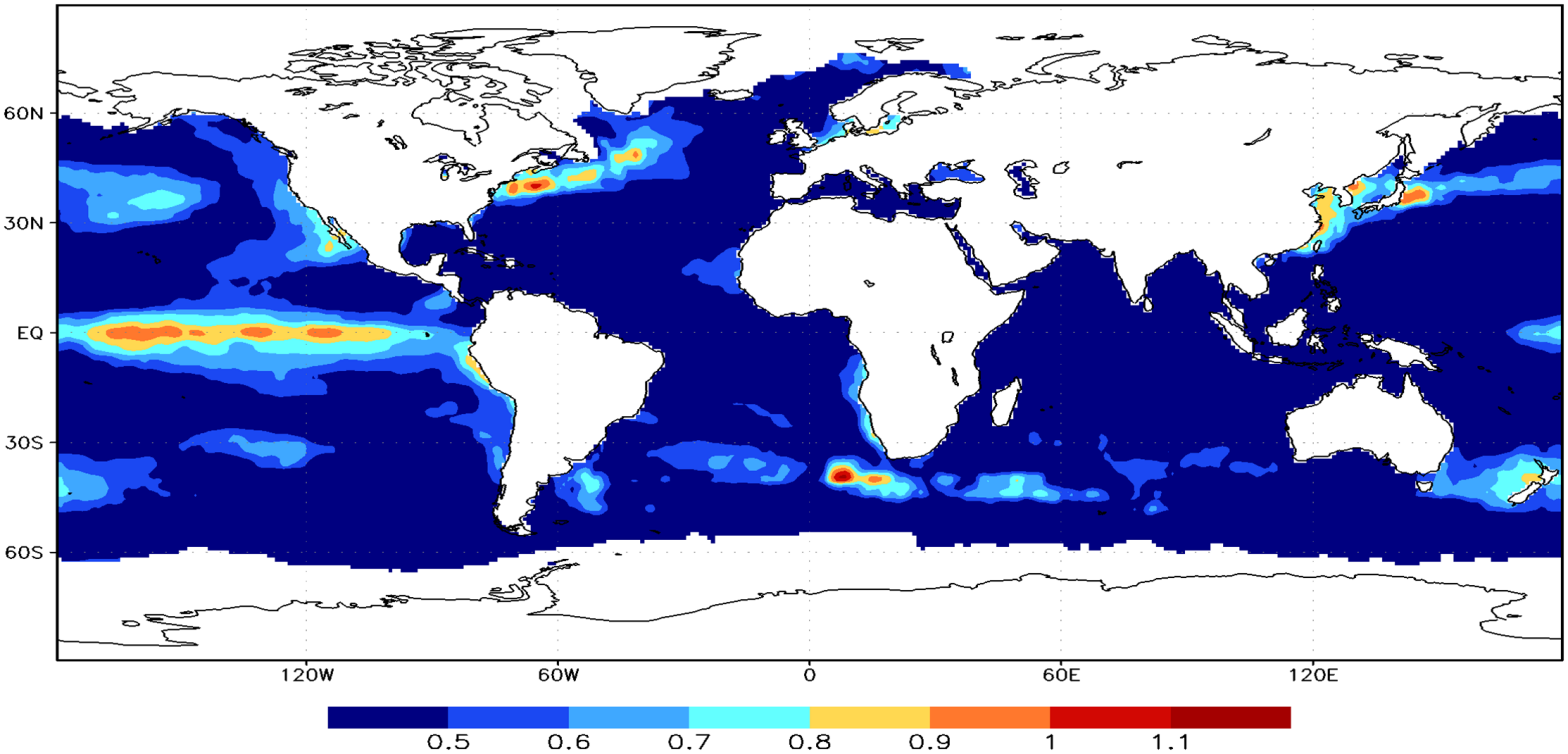
The geographical distributions of the index *χ*.

An index *χ* = *α* * *σ* (*α* represents the scaling exponent, *σ* represents the standard deviation) [46] was introduced to assess the variance extents of global SST. Jiang et al. [23] had applied the index to analyze the subarea characteristics of daily air temperature in China. We find that there exist obvious seesaw distributions for the index in the central and northern Pacific in Fig 4. This may imply an indicator on extreme climate events. The spatial distributions of the index χ is consistent with the geographical dependence of large standard deviation [34–35]. On the one hand, the index χ depends on the standard deviation to a large extent. On the other hand, the values of index χ are almost the same and 0.5 except for the regions of large standard deviation. The index χ may be an indicator of predictability [42]. The predictability of SST anomaly is low in those regions when the index χ is larger than 0.5, but high in other areas. The physical mechanisms need to be discussed in the future.

## Conclusions

In this study, we studied the global SST records for 139 years using the DFA method. The global SST variations during 1870–2009 are positively long-range correlated and can be characterized by power-law relationships. The mean slope values are 0.90 in the tropics, 0.87 in northern and southern hemisphere and the global zones, 0.84 in the intermediate latitudes of southern hemisphere and 0.81 over the intermediate latitude of northern hemisphere, respectively. The slope values depend on the geographical location of the SST time series.

By analyzing variations of the standard deviation and scaling exponents for the global SST anomaly records, we find that the values of standard deviation for SST variability are larger, the values of scaling exponents are smaller. In the meantime, large standard deviation can lead to increase the probability of occurrence of extreme value, accordingly, reduce the long-term persistence.

Furthermore, an index *χ* defined by the scaling exponent multiplies the standard deviation is used to analyze the variance extents of global SST. The spatial distributions where standard deviation of SST anomaly is large is consistent with that of the index χ. The index in the central and northern Pacific is higher than that in other places. This may provide a clue on the evaluation of predictability.

## References

[1] Peng CK, Buldyrev SV, Havlin S, Simons M, Stanley HE, Goldberger AL. Mosaic organization of DNA nucleotides[J]. Physical Review E, 1994, 49(2): 1685. 10.1103/physreve.49.1685 9961383

[2] Bunde A, Havlin S, Kantelhardt JW, Penzel T, Peter JH, Voigt K. Correlated and uncorrelated regions in heart-rate fluctuations during sleep [J]. Physical Review Letters, 2000, 85(17): 3736 11030994 10.1103/PhysRevLett.85.3736

[3] Kantelhardt JW, Koscielny-Bunde E, Rego HH, Havlin S, Bunde A. Detecting long-range correlations with detrended fluctuation analysis [J]. Physica A: Statistical Mechanics and its Applications, 2001, 295(3): 441–454.

[4] Hu K, Ivanov PC, Chen Z, Carpena P, Stanley HE. Effect of trends on detrended fluctuation analysis [J]. Physical Review E, 2001, 64(1): 011114. 10.1103/PhysRevE.64.011114 11461232

[5] Chen Z, Ivanov PC, Hu K, Stanley HE. Effect of nonstationarities on detrended fluctuation analysis [J]. Physical Review E, 2002, 65(4): 041107. 10.1103/PhysRevE.65.041107 12005806

[6] Kavasseri RG, Nagarajan R. A multifractal description of wind speed records [J]. Chaos, Solitons & Fractals, 2005, 24(1): 165–173.

[7] Telesca L, Lapenna V, Macchiato M. Multifractal fluctuations in seismic interspike series [J]. Physica A: Statistical Mechanics and its Applications, 2005, 354: 629–640.

[8] Koscielny-Bunde E, Bunde A, Havlin S, Goldreich Y. Analysis of daily temperature fluctuations [J]. Physica A: Statistical Mechanics and its Applications, 1996, 231(4): 393–396.

[9] Koscielny-Bunde E, Bunde A, Havlin S, Roman HE, Goldreich Y, Schellnhuber HJ. Indication of a universal persistence law governing atmospheric variability [J]. Physical Review Letters, 1998, 81(3): 729.

[10] Talkner P, Weber RO. Power spectrum and detrended fluctuation analysis: Application to daily temperatures [J]. Physical Review E, 2000, 62(1): 150. 10.1103/physreve.62.150 11088447

[11] Weber RO, Talkner P. Spectra and correlations of climate data from days to decades [J]. Journal of Geophysical Research: Atmospheres (1984–2012), 2001, 106(D17): 20131–20144.

[12] Bunde A, Havlin S. Power-law persistence in the atmosphere and in the oceans [J]. Physica A: Statistical Mechanics and its Applications, 2002, 314(1): 15–24.

[13] Govindan RB, Vyushin D, Bunde A, Brenner S, Havlin S, Schellnhuber HJ. Global climate models violate scaling of the observed atmospheric variability [J]. Physical Review Letters, 2002, 89(2): 028501 12097020 10.1103/PhysRevLett.89.028501

[14] Monetti RA, Havlin S, Bunde A. Long-term persistence in the sea surface temperature fluctuations [J]. Physica A: Statistical Mechanics and its Applications, 2003, 320: 581–589.

[15] Eichner JF, Koscielny-Bunde E, Bunde A, Havlin S, Schellnhuber HJ. Power-law persistence and trends in the atmosphere: A detailed study of long temperature records [J]. Physical Review E, 2003, 68(4): 046133. 10.1103/PhysRevE.68.046133 14683028

[16] Blender R, Fraedrich K. Long time memory in global warming simulations [J]. Geophysical Research Letters, 2003, 30(14).

[17] Kurnaz ML. Application of detrended fluctuation analysis to monthly average of the maximum daily temperatures to resolve different climates [J]. Fractals, 2004, 12(04): 365–373.

[18] Király A, Jánosi IM. Detrended fluctuation analysis of daily temperature records: Geographic dependence over Australia [J]. Meteorology and Atmospheric Physics, 2005, 88(3–4): 119–128.

[19] Liu Y, Cizeau P, Meyer M, Peng CK, Stanley HE. Correlations in economic time series [J]. Physica A: Statistical Mechanics and its Applications, 1997, 245(3): 437–440.

[20] Stanley HE, Amaral LAN, Goldberger AL, Havlin S, Ivanov PC, Peng CK. Statistical physics and physiology: monofractal and multifractal approaches [J]. Physica A: Statistical Mechanics and its Applications, 1999, 270(1): 309–324. 11543220 10.1016/s0378-4371(99)00230-7

[21] Vyushin D, Zhidkov I, Havlin S, Bunde A, Brenner S. Volcanic forcing improves atmosphere‐ocean coupled general circulation model scaling performance [J]. Geophysical research letters, 2004, 31(10).

[22] Varotsos C. Power‐law correlations in column ozone over Antarctica [J]. International Journal of Remote Sensing, 2005, 26(16): 3333–3342.

[23] Jiang L, Yuan NM, Fu ZT, Wang DX, Zhao X, Zhu XH. Subarea characteristics of the long-range correlations and the index χ for daily temperature records over China [J]. Theoretical and Applied Climatology, 2012, 109(1–2): 261–270.

[24] Jiang L, Li NN, Fu ZT, Zhang JP. Long-range correlation behaviors for the 0-cm average ground surface temperature and average air temperature over China [J]. Theoretical and Applied Climatology, 2015, 119(1–2): 25–31.

[25] Varotsos CA, Efstathiou MN, Cracknell AP. On the scaling effect in global surface air temperature anomalies [J]. Atmospheric Chemistry and Physics, 2013, 13(10): 5243–5253.

[26] Varotsos C, Efstathiou M, Tzanis C. Scaling behaviour of the global tropopause [J]. Atmospheric Chemistry and Physics, 2009, 9(2): 677–683.

[27] Zhu XH, Fraedrich K, Blender R. Variability regimes of simulated Atlantic MOC [J]. Geophysical research letters, 2006, 33(21).

[28] Gan Z, Yan Y, Qi Y. Scaling analysis of the sea surface temperature anomaly in the South China Sea [J]. Journal of Atmospheric and Oceanic Technology, 2007, 24(4): 681–687.

[29] Alvarez-Ramirez J, Alvarez J, Dagdug L, Rodriguez E, Echeverria JC. Long-term memory dynamics of continental and oceanic monthly temperatures in the recent 125 years [J]. Physica A: Statistical Mechanics and its Applications, 2008, 387(14): 3629–3640.

[30] Luo M, Leung Y, Zhou Y, Zhang W. Scaling Behaviors of Global Sea Surface Temperature [J]. Journal of Climate, 2015, 28(8): 3122–3132.

[31] Zhang WF, Zhao Q. Asymmetric long-term persistence analysis in sea surface temperature anomaly [J]. Physica A: Statistical Mechanics and its Applications, 2015, 428: 314–318.

[32] Rayner NA, Parker DE, Horton EB, Folland CK, Alexander LV, Rowell DP, et al Global analyses of sea surface temperature, sea ice, and night marine air temperature since the late nineteenth century [J]. Journal of Geophysical Research: Atmospheres (1984–2012), 2003, 108(D14).

[33] Fraedrich K, Blender R. Scaling of atmosphere and ocean temperature correlations in observations and climate models [J]. Physical Review Letters, 2003, 90(10): 108501 12689041 10.1103/PhysRevLett.90.108501

[34] McPhaden MJ. Genesis and evolution of the 1997–98 El Niño [J]. Science, 1999, 283(5404): 950–954. 9974381 10.1126/science.283.5404.950

[35] Lea DW, Pak DK, Spero HJ. Climate impact of late Quaternary equatorial Pacific sea surface temperature variations [J]. Science, 2000, 289(5485): 1719–1724. 10976060 10.1126/science.289.5485.1719

[36] Deser C, Alexander MA, Timlin MS. Understanding the persistence of sea surface temperature anomalies in midlatitudes [J]. Journal of Climate, 2003, 16(1): 57–72.

[37] Deser C, Alexander MA, Xie SP, Phillips AS. Sea surface temperature variability: Patterns and mechanisms [J]. Marine Science, 2010, 2. 10.1146/annurev-marine-120408-151453 21141660

[38] Knox RA, LUTHER DS, PHILANDER SGH. Estimates of equatorial upwelling between 1400 and 1100W during 1984 [J]. Journal of Geophysical Research, 1989, 94(C6): 8018–8020.

[39] Goswami BN, Madhusoodanan MS, Neema CP, Sengupta D. A physical mechanism for North Atlantic SST influence on the Indian summer monsoon [J]. Geophysical Research Letters, 2006, 33(2).

[40] Oppo DW, Rosenthal Y, Linsley BK. 2,000-year-long temperature and hydrology reconstructions from the Indo-Pacific warm pool [J]. Nature, 2009, 460(7259): 1113–1116. doi: 10.1038/nature08233 19713927 10.1038/nature08233

[41] Zhang Y, Ge E. Temporal scaling behavior of sea-level change in hong kong—Multifractal temporally weighted detrended fluctuation analysis [J]. Global and Planetary Change, 2013, 100: 362–370.

[42] Zhu X, Fraedrich K, Liu Z, Blender R. A demonstration of long-term memory and climate predictability [J]. Journal of Climate, 2010, 23(18): 5021–5029.

[43] Delworth T, Manabe S, Stouffer RJ. Interdecadal variations of the thermohaline circulation in a coupled ocean-atmosphere model [J]. Journal of Climate, 1993, 6(11): 1993–2011.

[44] Kerr RA. A North Atlantic climate pacemaker for the centuries [J]. Science, 2000, 288(5473): 1984–1985. 17835110 10.1126/science.288.5473.1984

[45] Frauen C, Dommenget D. Influences of the tropical Indian and Atlantic Oceans on the predictability of ENSO [J]. Geophysical Research Letters, 2012, 39(2).

[46] Chen X, Lin GX, Fu ZT. Long‐range correlations in daily relative humidity fluctuations: A new index to characterize the climate regions over China [J]. Geophysical research letters, 2007, 34(7).

